# Volumetric Ablation of Phenolic Resin in Extreme Environments: A Variable-Property Physics-Informed Neural Network Framework

**DOI:** 10.3390/polym18151855

**Published:** 2026-07-29

**Authors:** Wenqi Du, Te Ma, Hongwei Song

**Affiliations:** 1 Key Laboratory for Mechanics in Fluid Solid Coupling Systems, Institute of Mechanics, Chinese Academy of Sciences, Beijing 100190, China; duwenqi@imech.ac.cn (W.D.); songhw@imech.ac.cn (H.S.); 2 School of Engineering Science, University of Chinese Academy of Sciences, Beijing 100190, China

**Keywords:** physics-informed neural networks, pyrolysis, ablation behavior, variable properties, coupled PDEs

## Abstract

Phenolic resin ablation is a complex multi-physics process characterized by intense pyrolysis, strong thermal–chemical coupling, and drastic evolution of material properties. Predicting the through-thickness thermal response is computationally challenging, primarily due to the intricate coupling and the non-linear evolution of properties with temperature and pyrolysis. This study proposes a variable-property integrated Physics-Informed Neural Network (PINN) framework to model the high-temperature ablation of phenolic resin. Specifically, by constructing a loss function that strictly embeds the physics of heat conduction and pyrolysis, the framework explicitly captures the non-linear evolution of five thermophysical parameters, including thermal conductivity, specific heat, and density, throughout the ablation process. Numerical verifications across multiple scenarios demonstrate that the proposed variable-property PINN framework can simultaneously predict the sharp transient temperature and complex volumetric pyrolysis fields with a maximum relative error of less than 8.0% compared to the high-fidelity FEM baseline solutions. This mesh-free, efficient approach offers a novel paradigm for solving strongly coupled, variable-property ablation problems. It effectively overcomes the computational stiffness of conventional techniques, thereby providing valuable insights for the precise optimization of thermal protection systems.

## 1. Introduction

As typical thermosetting polymers, resin-based materials are widely employed as matrices in ablative composites due to their exceptional ablation resistance and low density, attracting significant attention for high-temperature aerospace applications [1,2,3]. In extreme high-temperature environments encountered by spacecraft during atmospheric re-entry or hypersonic vehicles during high-speed flight, resin-based materials achieve thermal protection via a sacrificial mechanism known as “volumetric ablation”. The ablation of resin-based materials is a complex, highly coupled multi-physics process. It encompasses heat transfer mechanisms (e.g., conduction and radiation) and mass transport phenomena (e.g., pyrolysis and charring), accompanied by irreversible evolution of the resin’s thermophysical and thermodynamic properties [4,5,6]. Therefore, developing a computational model that accurately characterizes volumetric ablation is pivotal. The model enables the precise resolution of the temperature field, degree of pyrolysis, and the coupled evolution of thermophysical properties. These insights are fundamental for predicting high-temperature mechanical performance and optimizing thermal protection structures.

Although traditional numerical methods, such as the Finite Element Method (FEM) and Finite Volume Method (FVM), are widely employed in ablation simulations, achieving precise solutions for the ablation of phenolic resins remains challenging. Fundamentally, ablation involves highly non-linear multi-physics coupling. Traditional methods, limited by their dependence on time-stepping discretization, require complex iterative schemes to handle the drastic mutations in material properties. Such a procedure necessitates strict convergence criteria to resolve the strong non-linearities, often leading to computational stiffness and algorithmic complexity, even in reduced-dimensional cases [7,8]. Furthermore, conventional grid discretization schemes severely degrade the prediction accuracy of key field variables like temperature [9,10,11]. Thirdly, thermophysical properties are nonlinearly dependent on temperature and the degree of pyrolysis. Traditional methods heavily rely on empirical formulas to fit the property evolution. Consequently, prediction errors escalate sharply once conditions exceed the applicable range of these empirical correlations. Specifically, the exponential sensitivity of Arrhenius-type reaction kinetics amplifies minor temperature fluctuations into drastic variations in source terms, often destabilizing standard solvers [12].

Addressing these limitations, neural network (NN) methods offer a robust solution for solving partial differential equations (PDEs) [13,14,15,16,17], owing to their exceptional ability to approximate arbitrary continuous functions. Specifically, Physics-Informed Neural Networks (PINNs) provide a novel approach for solving ablation problems of phenolic resin. PINNs directly solve partial differential equations by embedding physical laws, such as energy and mass conservation, into the loss function. PINNs operate within a mesh-free framework. Consequently, by eliminating the need for complex discretization, this approach effectively avoids the numerical instability typical of traditional grid-based methods. Furthermore, PINNs integrate highly non-linear characteristics, such as evolving thermophysical properties and moving boundaries, into the training process. This capability enables the precise and efficient determination of key ablation quantities. In addition, PINNs combine the dual advantages of data-driven and physics-constrained approaches [18,19,20,21,22,23,24]. By assimilating sparse experimental data to correct model deviations, they effectively bridge the gap between numerical simulations and actual operating conditions. Raissi et al. [25] introduced Physics-Informed Neural Networks (PINNs) to directly tackle non-linear problems. By utilizing automatic differentiation to compute derivatives, this method achieves precise PDE solutions at the training points. Moreover, PINNs act as surrogate models by treating analysis parameters as inputs. This capability supports real-time simulation, significantly accelerating the solution of complex non-linear problems.

Modeling phenolic resin ablation involves solving strongly non-linear PDEs. Under high heat flux, the material absorbs heat and heats up rapidly. These intense physicochemical reactions drive the volumetric pyrolysis process, leading to the generation of residual char. As the reaction deepens, key thermophysical properties (density, thermal conductivity, and specific heat capacity) display strong temperature-dependent characteristics. During ablation, the strong non-linear coupling among heat transfer, pyrolysis, and property evolution poses significant challenges for PINNs. Specifically, the ability to simultaneously satisfy physical laws and accurately capture the non-linear evolution of thermophysical properties remains an issue not fully addressed in current research. This study proposes a variable-property integrated Physics-Informed Neural Network (PINN) framework tailored for the high-temperature ablation of phenolic resin. Different from conventional approaches, our framework constructs a composite loss function that strictly embeds the physics of heat conduction and pyrolysis. This innovation enables the simultaneous and precise determination of the temperature field, pyrolysis degree, and the non-linear evolution of thermophysical parameters. Crucially, this is achieved without the need for complex iterative schemes. Ghaderi et al. [26] developed PINNs to model material pyrolysis, successfully solving for temperature and the degree of reaction. However, they simplified thermophysical properties as constants, neglecting their temperature dependence. This simplification leads to deviations from the actual ablation process. Niaki et al. [27] applied PINNs to simulate the thermochemical evolution of composite tooling systems during curing, accurately determining material temperature and the degree of cure at each instant. However, since the non-linearity of parameters in curing is significantly lower than in severe ablation, their method cannot be directly transferred to ablation problems. Liu et al. [28] established a PINN framework to identify the evolution of the ablation surface. By using temperature histories measured by thermocouples at various internal locations as inputs, they successfully reconstructed the temperature field and located the surface heat flux. However, they did not consider the feedback effect of thermophysical property evolution on the temperature field.

In summary, existing PINNs have proven feasible for solving PDEs in thermochemical processes. However, accurately incorporating the temperature-dependent thermophysical properties of phenolic resin remains a critical necessity. This integration is vital for resolving strongly coupled multi-physics interactions and ensuring high fidelity to the real ablation process. To address this, focusing on one-dimensional ablation, this paper proposes a PINN model that seamlessly integrates the non-linear evolution of these properties. The approach aims to overcome the computational bottlenecks of traditional methods, offering a novel paradigm for precisely predicting ablation behavior.

The ablation process of phenolic materials is governed by the heat conduction PDEs coupled with reaction kinetics equations. The proposed PINN achieves the solution to the ablation by formulating residuals based on the PDEs, initial conditions, and boundary conditions. Specifically, the non-linear dependencies of thermal conductivity, specific heat, and density on temperature and the degree of reaction are integrated into the loss function as physical constraints. Simultaneously, automatic differentiation is leveraged to accurately calculate the residuals of the heat transfer and reaction kinetics equations, ensuring that the PINN strictly adheres to the physical laws of the ablation process. The PINN prioritizes the solution of temperature and the degree of pyrolysis during phenolic ablation. Consequently, it achieves high precision and efficiency in determining five key physical quantities associated with the ablation process.

The physical model of the resin ablation process is initially established, providing explicit mathematical expressions for the heat conduction and reaction kinetics equations, as well as the initial and boundary conditions. Subsequently, the PINN solution framework is elaborated, highlighting the construction of a loss function utilizing automatic differentiation and multi-objective optimization. Ultimately, the model’s precision and effectiveness are validated against Finite Element Method (FEM) benchmarks under varying heat flux densities and different material systems.

## 2. The Physicochemical Process Description of Ablation

In high-temperature environments, phenolic resin undergoes bulk ablation. The decomposition of the resin absorbs heat, and the process is governed by the following heat conduction partial differential equation [27]:

(1) $$\frac{\partial }{\partial t}\left(\rho CT\right)=\frac{\partial }{\partial x}\left(k_{xx}\frac{\partial T}{\partial x}\right)+\frac{\partial }{\partial y}\left(k_{zz}\frac{\partial T}{\partial y}\right)+\frac{\partial }{\partial z}\left(k_{zz}\frac{\partial T}{\partial z}\right)+\dot{Q}$$

where T represents temperature, ρ,C,k are the density, specific heat capacity, and thermal conductivity of the material, $\dot{Q}$ is the heat source term arising from the thermal decomposition of phenolic resin. It can be expressed as a function of the degree of reaction and temperature. The functional relationship is given by

(2) $$\dot{Q}=H_{v}\frac{d\alpha }{dt}$$

where $H_{v}$ denotes the heat of reaction per unit mass. During the ablation process, the evolution of the degree of pyrolysis α is typically governed by a reaction kinetics equation, which describes the relationship between the degree of reaction and the instantaneous temperature [27].

(3) $$\frac{d\alpha }{dt}=Ae^{-E_{a}/RT}{\left(1-\alpha \right)}^{N},A=J_{0}/\rho$$

where *A* is the reaction coefficient, *E_a_* denotes the apparent energy of activation, *R* is the universal gas constant, *N* is the reaction order, *J*_0_ is the pre-exponential factor.

It should be emphasized that the local solid mass loss effect is fully accounted for in the present continuum framework through the transient evolution of the bulk density. The time-dependent mass depletion rate, governed by the Arrhenius kinetic rate, directly scales the endothermic pyrolysis energy sink. However, the internal pyrolysis gas transport within the decomposing matrix is simplified. Specifically, the pressure accumulation of the trapped gaseous species, Darcy-like mass flux percolation, and the resulting gas–solid convective heat transfer term are neglected. This formulation operates under the assumption that the volatile gases escape through the crack-permeable char network rapidly, and that the convective cooling contribution remains secondary to the dominant conductive thermal wave and localized decomposition chemical enthalpy under the simulated extreme heat fluxes. This standard simplification ensures that the PINN model can focus efficiently on resolving the severe computational stiffness induced by state-dependent solid physical property variations.

**Figure 1:**
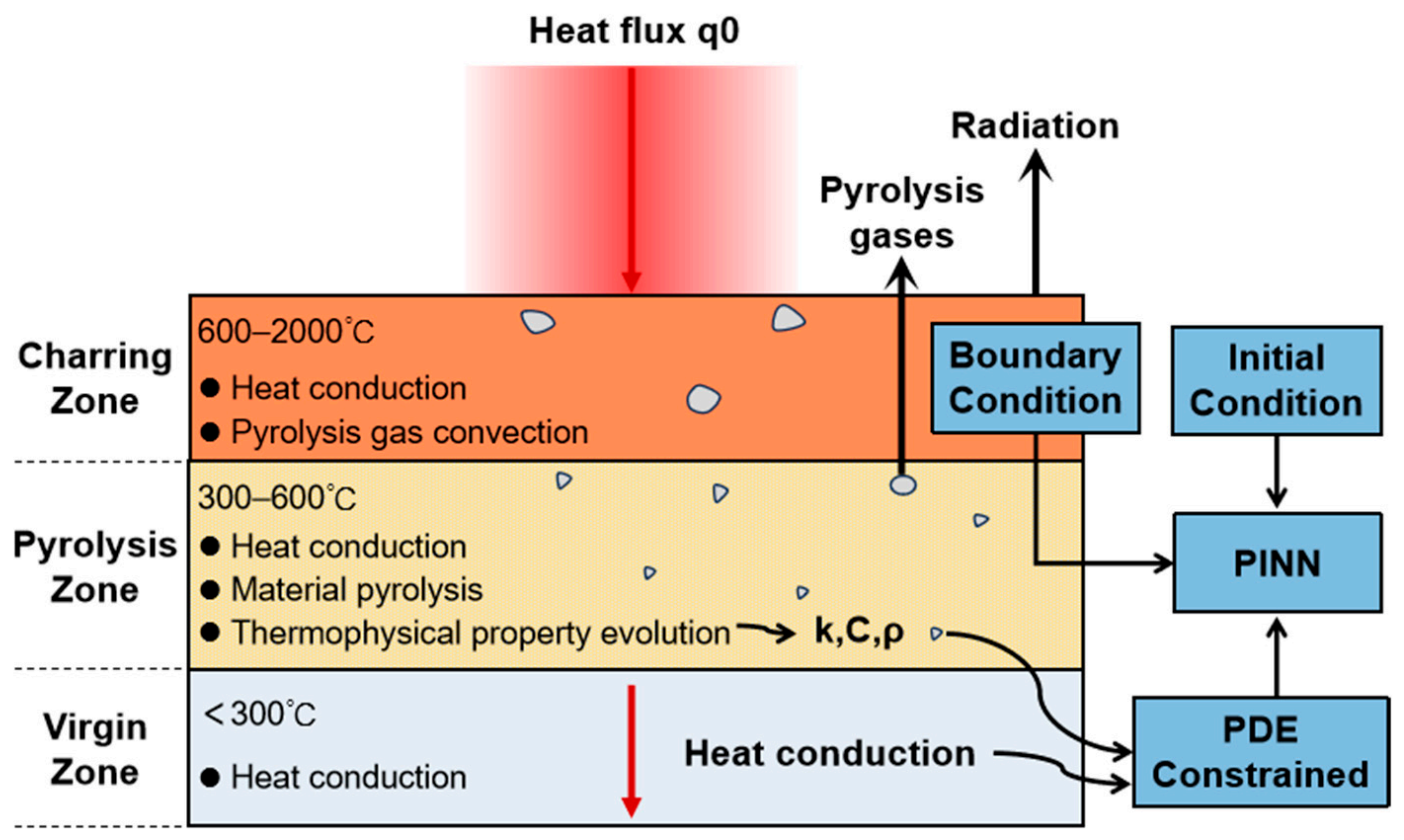
The process of ablation.

Under conditions of transient and severe ablation, the pyrolysis of phenolic resin and subsequent char formation lead to a dynamic evolution of thermophysical properties (density, specific heat, and thermal conductivity), which vary with time, temperature, and the extent of reaction. To visually represent the underlying multi-phase degradation process, Figure 1 provides a schematic of the material state under high-power thermal loading. As illustrated, the material domain is naturally segmented into three distinct physical zones based on the local degree of pyrolysis (α): Virgin Zone (α = 0): The pristine, unreacted composite matrix where temperatures remain below the decomposition threshold. Pyrolysis/Reaction Zone (0 < α < 1): The highly active chemical degradation front characterized by steep temperature gradients, active resin mass loss, and endothermic volatilization. Char Zone (α = 1): The fully carbonized, porous residue matrix consisting entirely of the solid charred phase after complete resin depletion. This continuous α-based zoning forms the physical foundation for the spatially varying thermodynamic properties evaluated in our PINN and FEM models. The thermophysical properties are expressed as functions of temperature and the extent of reaction [29]:

(4) $$C=\left(1-\alpha \right)C_{b}+\alpha C_{p},C_{b}=\left(M_{Cb}+N_{Cb}T\right)C_{b0},C_{p}=\left(M_{Cp}+N_{Cp}T\right)C_{p0}$$

(5) $$k=\left(1-\alpha \right)k_{b}+\alpha k_{p},k_{b}=\left(M_{kb}+N_{kb}T\right)k_{b0},k_{p}=\left(M_{kp}+N_{kp}T\right)k_{p0}$$

(6) $$\rho =\left(1-\alpha \right)\rho_{b}+\alpha \rho_{p}$$

where k,C,ρ denote the specific heat, thermal conductivity, and density, with subscripts *b* and *p* representing the virgin and char states, respectively. The coefficients *M* and *N* $\left(e.g.,\;M_{Cp},N_{Cp},M_{kp},N_{kb},M_{\rho p},N_{\rho b}\right)$ define their dependency on temperature. Therefore, considering the phenolic resin ablation with temperature-dependent thermophysical properties, it is necessary to solve the strongly non-linear differential equations involving the mutual coupling of temperature, degree of pyrolysis, and thermophysical parameters.

For the ablation process, a constant heat flux is applied at the boundary. Taking thermal radiation into account, this configuration satisfies the boundary condition of the second kind. The energy balance between the boundary heat flux and the internal heat conduction is governed by the following equation:

(7) $$-k_{xx}{\left.\frac{\partial T}{\partial x}\right|}_{x=L}=q-\varepsilon \sigma \left(T_{s}^{4}-T_{\infty }^{4}\right)$$

where *q* denotes the constant heat flux density, $T_{s}$ represents the material surface temperature, $T_{\infty }$ is the ambient temperature, ε is the emissivity, and σ is the Stefan–Boltzmann constant. In this study, the emissivity ε is prescribed as 0.85 based on typical characterizations of phenolic, and the ambient temperature is maintained at $T_{\infty }$ = 300 K. These parameters complete the non-linear Robin boundary constraint imposed on the continuous PINN loss graph at the forward boundary.

The mathematical framework developed in this study utilizes a one-dimensional (1D) transient formulation along the through-thickness direction. This dimensional simplification is physically justified under specific extreme environmental and geometric configurations typical of thermal protection system (TPS) testing:

(1) The 1D approximation assumes that the characteristic heating diameter D is significantly larger than the material thickness L. Under this condition, edge effects and lateral heat losses are negligible in the core region, rendering the thermal wave propagation strictly perpendicular to the surface.

(2) Under ultra-high heat fluxes, the intense surface heating establishes a severe temperature gradient along the depth, which is orders of magnitude greater than any unintended in-plane lateral gradients. Consequently, the net thermal energy transport is overwhelmingly dominated by through-thickness conduction and localized endothermic pyrolysis.

(3) Since the object of this study is pure, unreinforced phenolic resin, the material exhibits macroscopically isotropic thermal behavior. This eliminates preferential lateral conduction pathways that typically occur in directionally reinforced composites, further ensuring that a 1D continuum model can accurately capture the transient temperature and decomposition profiles.

Therefore, while a multi-dimensional model would be required for highly focused or complex localized heat sources, the present 1D variable-property PINN framework provides a rigorous, highly efficient, and high-fidelity representation for standard large-area extreme thermal ablation problems.

The initial condition is expressed as

(8) $$\begin{array}{c} {\left.T\right|}_{t=0}=T_{0}\left(x\right)\\ {\left.\alpha \right|}_{t=0}=\alpha_{0}\left(x\right)\\ {\left.k\right|}_{t=0}=k_{0}\left(x\right)\\ {\left.C\right|}_{t=0}=C_{0}\left(x\right)\\ {\left.\rho \right|}_{t=0}=\rho_{0}\left(x\right) \end{array}$$

where *T* represents the initial temperature, α denotes the initial degree of reaction for the resin, which is set to 0 before the onset of ablation. k,C,ρ represent the initial thermophysical properties of the phenolic resin, respectively.

## 3. Physics-Informed Neural Network for Ablation

In this section, we establish a Physics-Informed Neural Network (PINN) framework for the one-dimensional ablation problem. We solve the governing partial differential equations to ensure the stable convergence of the PINN solver.

### 3.1. PINN for Ablation

**Figure 2:**
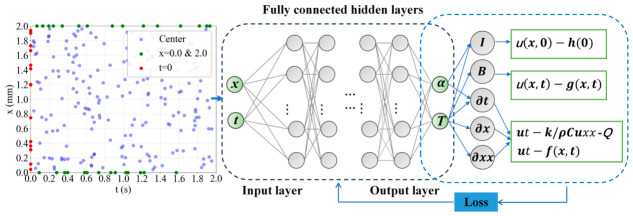
General architecture of the PINN framework.

In this PINN model, the input represents the spatial and temporal coordinates (*x*, *t*), while the output comprises the temperature, reaction extent, and thermophysical properties (*T*, *α*, *ρ*, *C*, *k*). Within this framework, a feedforward multilayer neural network is utilized to approximate these multiple variables (*T*, *α*, *ρ*, *C*, *k*).

(9) $$y\left(x,t\right)\approx f\left(x;t;\theta \right)$$

where *f* is a multilayer neural network parameterized by *θ*. The architecture of the constructed PINNs model is illustrated in Figure 2, where *u*(x, 0) − *h*(0) is the initial condition residual, *u*(*x*, t) − *g*(*x*, t) is the boundary condition residual, *u*t − *k*/*ρCuxx* − *Q* and *u*t − *f*(*x*, t) is the governing partial differential equation residual. The architecture of the constructed PINN model is illustrated in Figure 2. The established PINNs are trained by minimizing the residuals at the collocation points *X* to update the network parameters *θ*.

(10) $$\theta^{*}=\mathrm{argmin}L_{f}\left(X;\theta \right)$$

The network corresponds to the calculation process described by

(11) $$f\left(x;t;\theta \right)=\sum^{n}\circ \sigma \circ \sum^{n-1}\circ \ldots \circ \sigma \circ \sum^{1}\left(x\right)$$

where

(12) $$\sum^{d}\left(Z^{d-1}\right)=W^{d}\cdot Z^{d-1}+b^{d},\mathrm{where}\;d=1,\ldots ,n$$

An n-layer multilayer feedforward neural network was constructed, consisting of an input layer, n − 1 hidden layers, and an output layer. In Equation (11), the symbol ∘ denotes the composition operation. $Z^{d}$ represents the network output vector (*T*, *α*, *ρ*, *C*, *k*), while $Z^{d-1}$ signifies the network input vector (*x*,*t*). Furthermore, $W^{d}$ and $b^{d}$ denote the neuronal weights and biases within the d-layer network architecture, respectively.

A non-linear activation function, σ, is applied to the mapped output of each layer $\sum^{n}$. The activation function is instrumental in modeling the complex non-linear mapping from inputs to outputs. It constitutes a hyperparameter that directly governs the convergence and accuracy of the solution. The training performance of PINNs is significantly influenced by the activation function. Consequently, activation functions such as ReLU, Sigmoid, and Tanh are frequently utilized. Among these, ReLU is non-differentiable at zero due to its linear nature, and with its second-order derivative constantly zero, it fails to support the higher-order derivative computations essential for solving partial differential equations. In contrast, Tanh, being a smooth activation function, effectively supports the computation of higher-order derivatives required by the governing equations in ablation problems while also possessing the desirable property of non-vanishing gradients. These characteristics render it more suitable for neural network frameworks that incorporate physical laws. Otherwise, considering the non-linearity coupling T and α in the ablation process, the Tanh is preferred due to its smoothness and non-vanishing gradients. These properties are critical for accurately capturing the non-linear dynamics and ensuring robust convergence. Based on the above analysis, Tanh is selected as the activation function for the hidden layers in this study, as its smoothness enhances the regularization effect of PINNs and enables a more accurate evaluation of the model’s generalization error.

(13) $$\tanh\left(x\right)=\frac{e^{x}-e^{-x}}{e^{x}+e^{-x}}$$

The degree of pyrolysis, *α*, represents the normalized progress of decomposition and is physically bounded within the interval (0, 1). To satisfy this constraint, the Sigmoid activation function is employed for the output node corresponding to *α*. The Sigmoid function is defined as

(14) $$Sigmoid\left(x\right)=\frac{1}{1+e^{-x}}$$

The choice of activation functions within the variable-property PINN framework is customized to accommodate both higher-order numerical differentiability and explicit domain boundary constraints. Specifically, the Tanh function is allocated to the hidden layers governing the dimensionless temperature output, while the Sigmoid function parameterizes the final output layer of the pyrolysis degree α. To justify this architectural configuration, a brief algorithmic sensitivity evaluation was performed against other baseline activation function pairings (e.g., ReLU and Swish). Standard piecewise linear activations like ReLU yield non-convergent solutions because their second-order derivatives vanish identically across the continuous domain, rendering the backpropagation of the conductive PDE loss impossible. On the other hand, while modern smooth activations like Swish maintain gradient continuity, they lack an upper/lower mathematical bound. Given that the chemical pyrolysis degree is strictly bounded by physical definition (α ∈ [0, 1]), enforcing a Sigmoid profile acts as a hard structural inductive bias. Comparative tests reveal that substituting Sigmoid with unconstrained activations (such as Swish or Tanh) causes the unlabeled training points to transiently drift into non-physical regimes (α < 0 or α > 1) during early epoch stabilization, increasing the maximum relative error to over 14%. Consequently, the decoupled Tanh-Sigmoid architecture guarantees optimal compliance with both the smoothness required by automatic differentiation and the mathematical bounds dictated by mass conservation.

In the formulation of the loss function, the partial derivatives of the variables are computed via automatic differentiation [8,13]. Regarding data configuration for network training, this paper employs a random sampling strategy, setting 20,000 training points within the solution domain, and simultaneously selecting 2000 sampling points at the spatial boundary x = L and the initial condition t = 0 to apply boundary and initial constraints. The constructed PINN model consists of a fully connected neural network with five hidden layers, each containing 128 neurons. Collocation point and network architecture independence study result is shown in Table A1. Model training utilizes the Adam optimizer with a learning rate of 0.001 and 20,000 iterations. All solutions are implemented in a Python 3.9 environment using the PyTorch v2.7.0 framework, running on a workstation equipped with an Intel Core i7-6800K CPU (3.40 GHz), 64 GB of memory, and a Windows 10 operating system.

### 3.2. Nondimensionalization of the PDEs

The presence of exponential terms in the pyrolysis kinetics introduces numerical instability. Specifically, large variations in temperature inputs can lead to exploding values for the calculated degree of pyrolysis, ultimately rendering the loss function incapable of convergence. To prevent optimization divergence caused by the massive scale differences between spatial domains (10^−3^ m), time domains (10 s), and temperatures (10^3^ K), we introduced a strict scaling procedure. We define dimensionless coordinates $\bar{x}=\frac{x}{x^{*}},\bar{y}=\frac{y}{y^{*}}$, and dimensionless temperature $\bar{T}=\frac{T}{T^{*}}$. The degree of pyrolysis $\bar{\alpha }=\frac{\alpha }{\alpha^{*}}$ remains bounded within [0,1] and requires no further scaling. This maps all input and target fields precisely within an order of magnitude of unity, ensuring stable gradient propagation. The nondimensionalization process is as follows:

(15) $$\bar{t}=\frac{t}{t^{*}},\bar{x}=\frac{x}{x^{*}},\bar{y}=\frac{y}{y^{*}},\bar{T}=\frac{T}{T^{*}},\bar{\alpha }=\frac{\alpha }{\alpha^{*}}$$

$t^{*},x^{*},y^{*},T^{*},\alpha^{*}$, where all are dimensionless quantities. Thus, Equation (1) can be rewritten as

(16) $$\begin{array}{c} \frac{\rho_{0}C_{0}T_{0}}{t_{ref}}\frac{\partial \left(\rho^{*}C^{*}T^{*}\right)}{\partial t^{*}}=\frac{k_{0}T_{0}}{L^{2}}\frac{\partial }{\partial x^{*}}\left(k^{*}\frac{\partial T^{*}}{\partial x^{*}}\right)+\frac{Q_{0}\rho_{0}}{t_{ref}}\rho^{*}\frac{d\alpha^{*}}{dt^{*}}\\ \frac{\rho_{0}C_{0}L^{2}}{k_{0}t_{ref}}\frac{\partial \left(\rho^{*}C^{*}T^{*}\right)}{\partial t^{*}}=\frac{\partial }{\partial x^{*}}\left(k^{*}\frac{\partial T^{*}}{\partial x^{*}}\right)+\frac{Q_{0}}{C_{0}T_{0}}\rho^{*}\frac{d\alpha^{*}}{dt^{*}} \end{array}$$

The kinetics of the pyrolysis reaction are described by the following equation:

(17) $$\frac{d\alpha^{*}}{dt^{*}}=\frac{\rho_{0}C_{0}L^{2}}{k_{0}}A\exp\left(-\frac{E_{a}}{RT_{0}T^{*}}\right)$$

### 3.3. Loss Function Formulation

In the PINNs framework, the loss function is formulated based on the governing PDEs describing the ablation process, such as heat conduction and pyrolysis kinetics. specifically, these PDEs are first converted into their nondimensionalized forms to ensure numerical stability. The physics-informed loss is then defined by calculating the residuals of these dimensionless PDEs, utilizing the network’s outputs and their corresponding partial derivatives computed via automatic differentiation. The total loss function is defined as the sum of three components: the loss derived from the governing PDEs, the initial condition loss, and the boundary condition loss.

(18) $$L_{total}=L_{\alpha 0}+L_{T0}+L_{bc}+L_{T}+L_{\alpha }$$

The loss function is calculated at all training points. Based on the heat conduction PDE with an internal heat source term and the pyrolysis reaction kinetic equation during the ablation process, the loss function is formulated as follows:

(19) $$\begin{array}{c} L_{T}=\frac{1}{n}\sum_{i=1}^{n}{\left({\left.\frac{\partial T}{\partial t}\right|}_{x=i}-{\left.\frac{k_{xx}}{\rho C}\frac{\partial^{2}T}{\partial x^{2}}\right|}_{x=i}-{\left.\frac{\rho_{0}Q_{r}}{\rho C}\frac{d\alpha }{dt}\right|}_{x=i}\right)}^{2}\\ L_{\alpha }=\frac{1}{n}\sum_{i=1}^{n}{\left({\left.\frac{\partial \alpha }{\partial t}\right|}_{x=i}-{\left.\frac{A}{\rho }\exp\left(-E_{a}/RT\right){\left(1-\alpha \right)}^{N}\right|}_{x=i}\right)}^{2} \end{array}$$

where *n* denotes the number of training points, i∈x. The dependence of the material properties on temperature and the degree of pyrolysis is given by Equations (4)–(6).

For the one-dimensional ablation problem, the loss functions for the initial and boundary conditions are defined as follows:

(20) $$\begin{array}{c} L_{\alpha 0}=\frac{1}{n}\sum_{i=1}^{n}{\left({\left.\alpha \right|}_{t=0}-\alpha_{0}\left(x\right)\right)}^{2}\\ L_{T0}=\frac{1}{n}\sum_{i=1}^{n}{\left({\left.T\right|}_{t=0}-T_{0}\left(x\right)\right)}^{2}\\ L_{T\_bc}=\frac{1}{n}\sum_{i=1}^{n}{\left(q-{\left.k\frac{\partial T}{\partial x}\right|}_{x=0}\right)}^{2} \end{array}$$

Specifically, a constant heat flux *q* is imposed at x=l.

**Figure 3:**
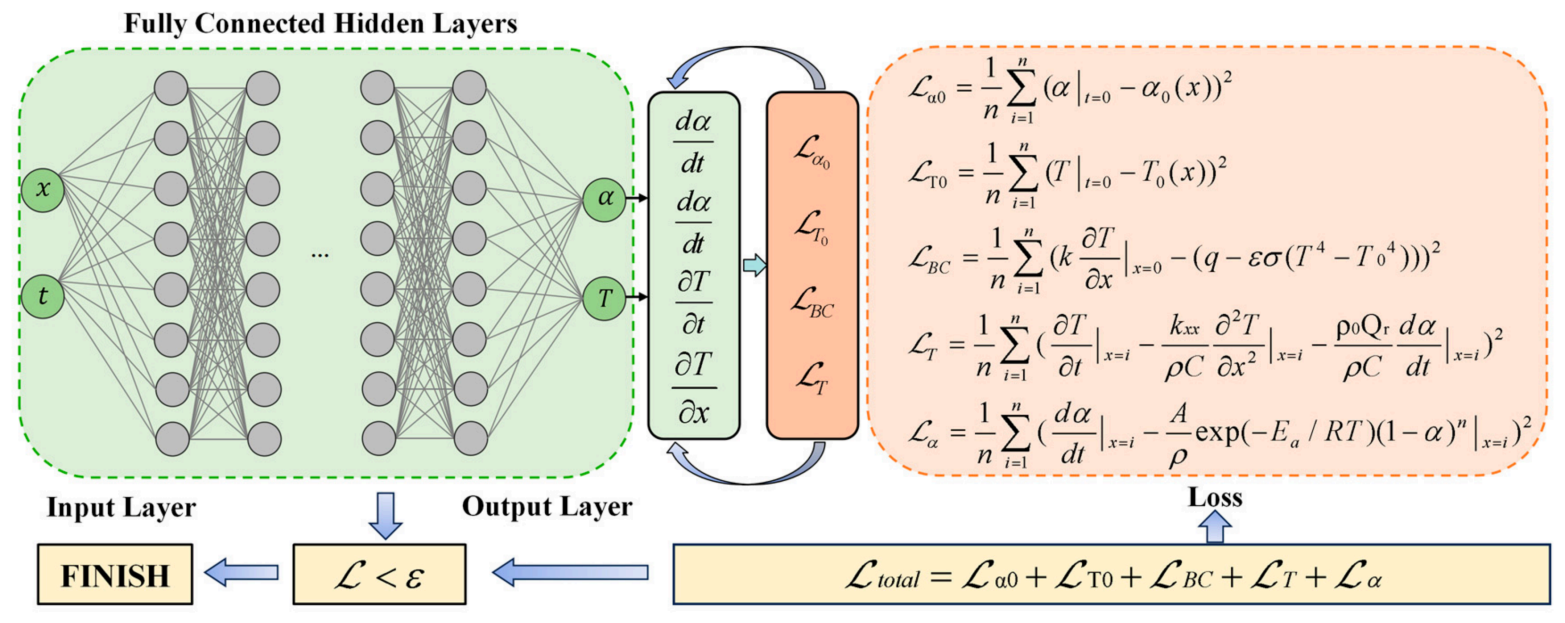
Schematic of the loss function formulation of PINN.

Figure 3 illustrates the overall architecture of the proposed PINNs and the mechanism for embedding physical laws. The framework seamlessly integrates the heat conduction and pyrolysis equations governing the ablation process into the network’s optimization objective. Specifically, taking spatio-temporal coordinates (*x*, *t*) as inputs, the network predicts the physical fields via fully connected layers and is trained by minimizing the loss function.

### 3.4. Adaptive Weighting Strategy

In addition to the construction of the loss function, the convergence balance during optimization requires careful consideration. The inherent scale disparities among different physical constraint terms, such as PDE residuals, boundary conditions, and initial conditions, coupled with the inconsistent convergence rates of the temperature and pyrolysis fields, can bias the optimization process toward specific loss components. To equilibrate the contribution of each term to parameter updates, an adaptive weighting strategy is adopted in this work, formulating the total loss function as a weighted sum:

(21) $$L\left(\theta \right)=\lambda_{PDE}L_{PDE}+\lambda_{0}L_{0}+\lambda_{BC}L_{BC}$$

where $\lambda_{i}$ are adaptable hyperparameters. Unlike static hyperparameters, the weighting coefficients are dynamically modulated based on gradient statistics during training. This mechanism harmonizes conflicting loss components, ensuring simultaneous satisfaction of boundary constraints and governing equations, thereby significantly enhancing predictive fidelity for strongly coupled non-linear problems.

(22) $${\hat{\lambda }}_{i}=\frac{\max\theta \left\|\nabla_{\theta }L_{ref}\right\|}{\mathrm{mean}\theta \left\|\nabla_{\theta }L_{i}\right\|}$$

To prevent excessive stochastic oscillations in the loss landscape and ensure a smooth trajectory for the Adam optimizer, a temporal filtering protocol utilizing an exponential moving average (EMA) is introduced. The actual operational weight is updated at a prescribed frequency of every k = 10 iterations. The memory decay factor is tightly bounded at 0.1. The initial condition weight follows an identical adaptive tracking and execution timeline. The primary mathematical and physical impacts of this adaptive mechanism on network convergence are twofold:

In early training phases, boundary constraints exhibit intense spatial gradients due to external thermal loads (e.g., localized flux or radiation). Static uniform networks inherently suffer from severe gradient starvation at boundaries because the dense spatio-temporal domain elements inside the PDE loss overwhelm the backpropagation signal. The adaptive scheme scales up dynamically by up to 2–3 orders of magnitude during these critical epochs, ensuring boundaries are strictly respected.

By maintaining a normalized gradient magnitude across all loss graphs, the optimizer avoids getting trapped in unphysical local minima. Empirical validation shows that while fixed-weight training stalls at a global loss plateau of *L* ≈ 10^−2^ with visible boundary violations, the adaptive loss-weighting strategy drives the compound convergence curve smoothly down to *L* ≤ 10^−5^, securing a tenfold acceleration in early-stage training efficiency.

## 4. Results

The training dataset is constructed using a random sampling strategy. To evaluate the capability of the proposed PINN in solving the complex multi-physics fields involved in the ablation process, this study conducts a comparative numerical verification by benchmarking the PINN solutions against finite element results across four distinct material systems and thermal loading conditions. Specifically, Cases 1 and 2 utilize the Phenolic I, while Cases 3 and 1-A investigate the Phenolic II, as shown in Table 1. To maximize physical fidelity, the computational framework enforces surface thermal radiation boundaries, sets a uniform initial temperature of 300 K, and integrates the fully coupled and temperature-dependent non-linearities of the thermophysical properties, and the parameters of phenolic ablation model are shown in Table 2.

### 4.1. Finite Element Method Solution of the Ablation Process

To evaluate the predictive accuracy of the proposed PINN, a Finite Element Method (FEM) simulation was established using the commercial software Abaqus 2021/Standard as the baseline ground truth. The 3D computational domain, defined as x in [0, 2 mm], the element type is DC3D8 with a global mesh size of 0.1 mm. The mesh independence verification of FEM is shown in Table A2 of Appendix A. The internal heat conduction exhibited a strictly one-dimensional heat transfer characteristic along the thickness direction. This behavior is physically attributed to the uniform heat flux fully covering the top surface and the assumption of adiabatic boundary conditions on the other surfaces. Based on this physical symmetry, the transient temperature field and other data extracted along the central axis of the 3D model are mathematically equivalent to the 1D governing equations. We use the 3D model serve as a reliable ground truth for validating the predictive accuracy of the PINN.

**Figure 4:**
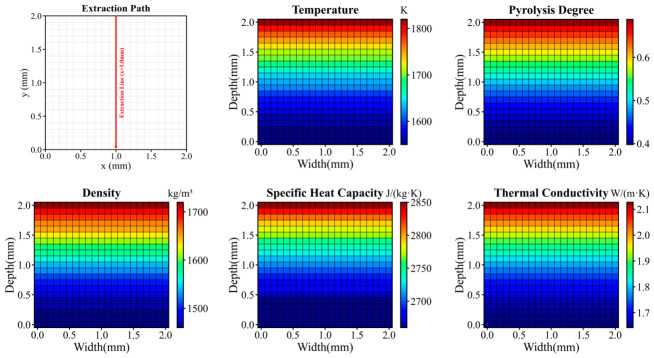
FEM simulation for the heat flux of 100 W/cm^2^ condition at t = 10 s.

The material properties, including temperature-dependent thermal conductivity and specific heat capacity, were defined through the UMATHT user subroutine to account for complex pyrolysis and ablation behaviors. The initial uniform temperature of the domain was set to T_0_ = 300 K and α = 0. For the boundary conditions, a heat flux was applied at the front surface x = 2 mm using the surface heat flux definition, while the rear surface x = 0 was assumed to be adiabatic. The transient thermal response was computed over a total time of t = 10 s. To ensure the accuracy and stability of the numerical solution, the time step is 2 s. As illustrated in Figure 4, the contour plot of the FEM solution is presented at 10 s under a heat flux of 100 W/cm^2^.

### 4.2. Numerical Verification Under Multiple Conditions

**Case 1: Ablation Process of Type I Phenolic Material (31 W/cm^2^, N = 1)**

**Figure 5:**
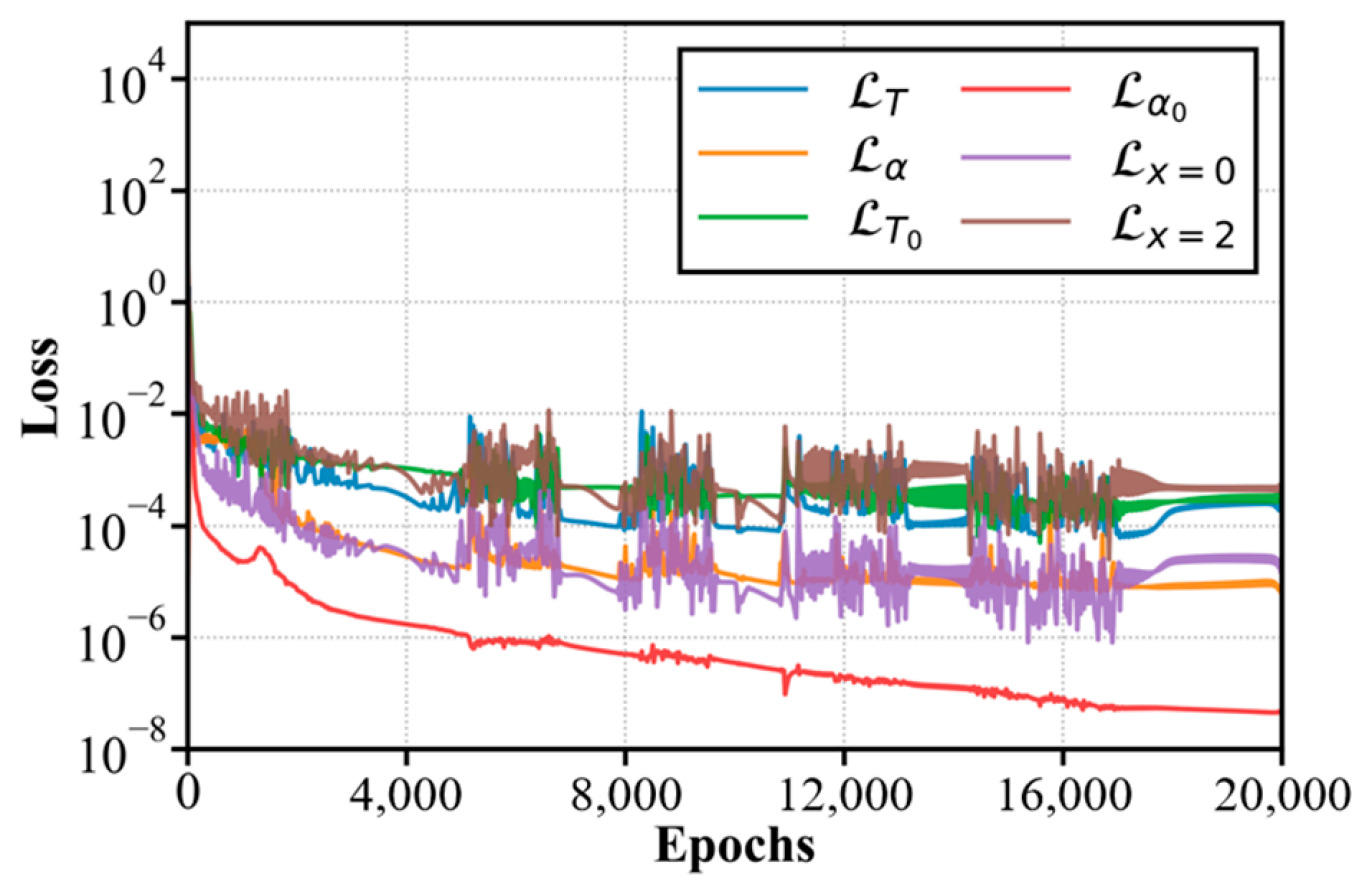
Loss function curves for Case 1.

As the initial numerical verification of the proposed computational framework, Case 1 investigates volumetric ablation under a heat flux of 31 W/cm^2^. Figure 5 illustrates the training process, demonstrating the stable and monotonic convergence of the PINN loss function. All loss components, including initial conditions, boundary conditions, and PDE residuals governing heat transfer and pyrolysis kinetics, exhibit progressive convergence throughout the training process, eventually settling in the range of 10^−3^~10^−8^ after 8000 epochs.

**Figure 6:**
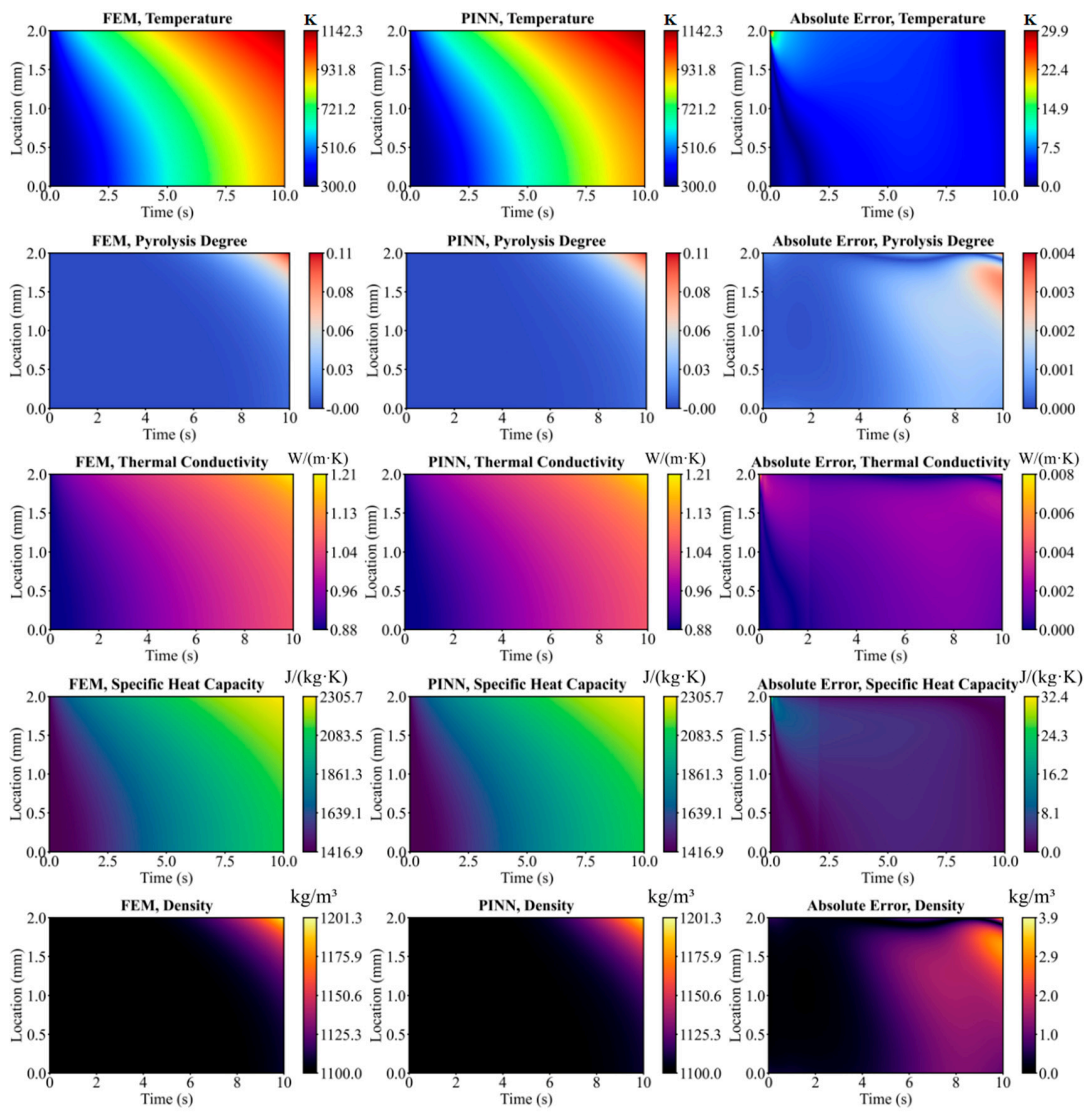
Comparison between PINN and FEM solutions (q = 31 W/cm^2^).

Figure 6 illustrates the predicted temporal evolution of temperature, degree of pyrolysis, and the corresponding thermophysical properties throughout the ablation process. A comprehensive analysis of Case 1 reveals excellent agreement between the PINN predictions and the FEM solutions. Even in high-temperature regions characterized by intense reactions, the relative errors for temperature, degree of pyrolysis, and thermophysical properties remain below 5%. These results demonstrate that the PINN framework accurately simulates the volumetric ablation of phenolic resin, exhibiting superior stability and precision in handling complex physical mechanisms, including constant heat flux, boundary radiation, and exothermic ablation reactions.

**Figure 7:**
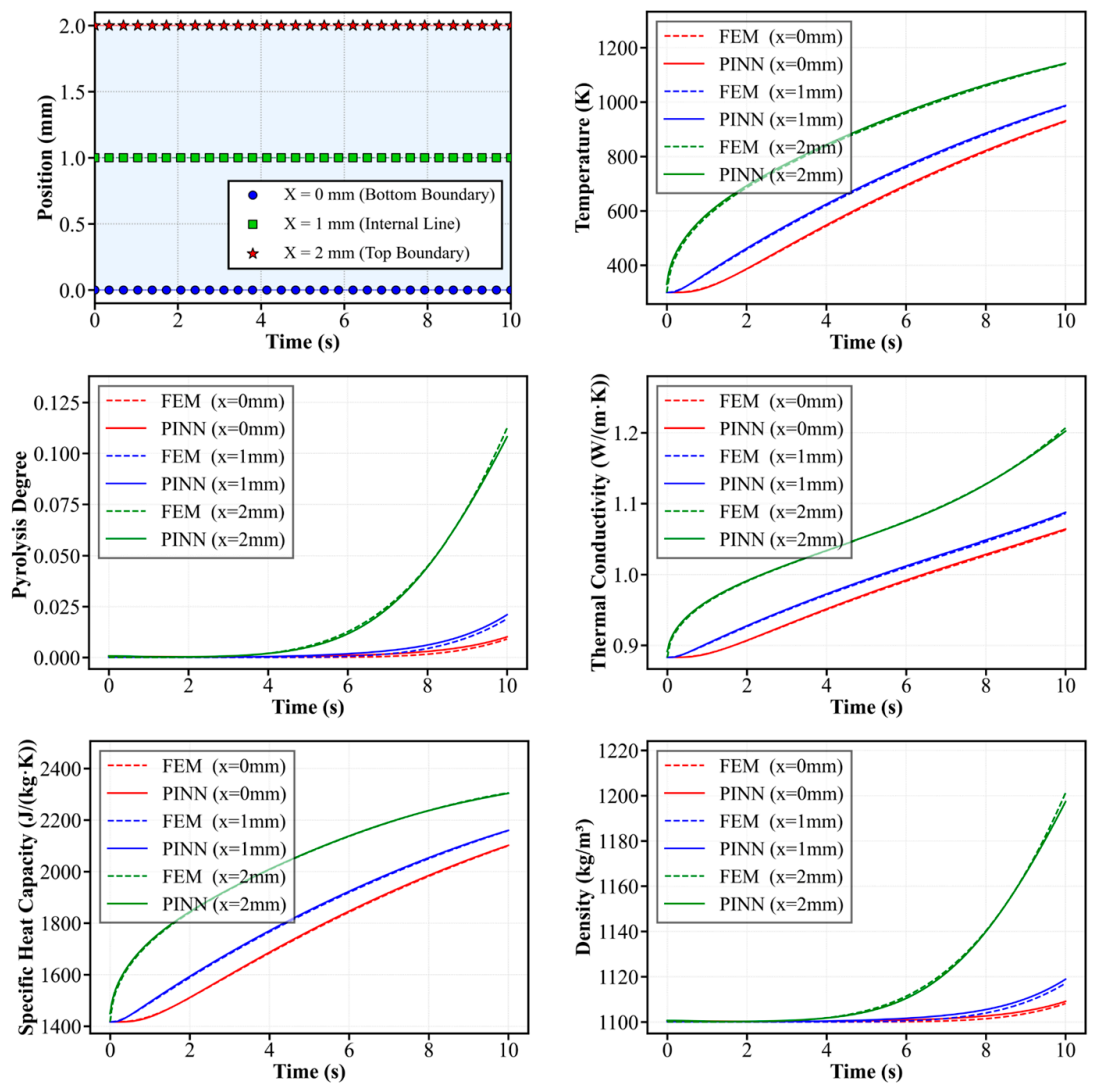
Comparison of PINN and FEM results at different locations (x = 0, 1, 2 mm).

**Figure 8:**
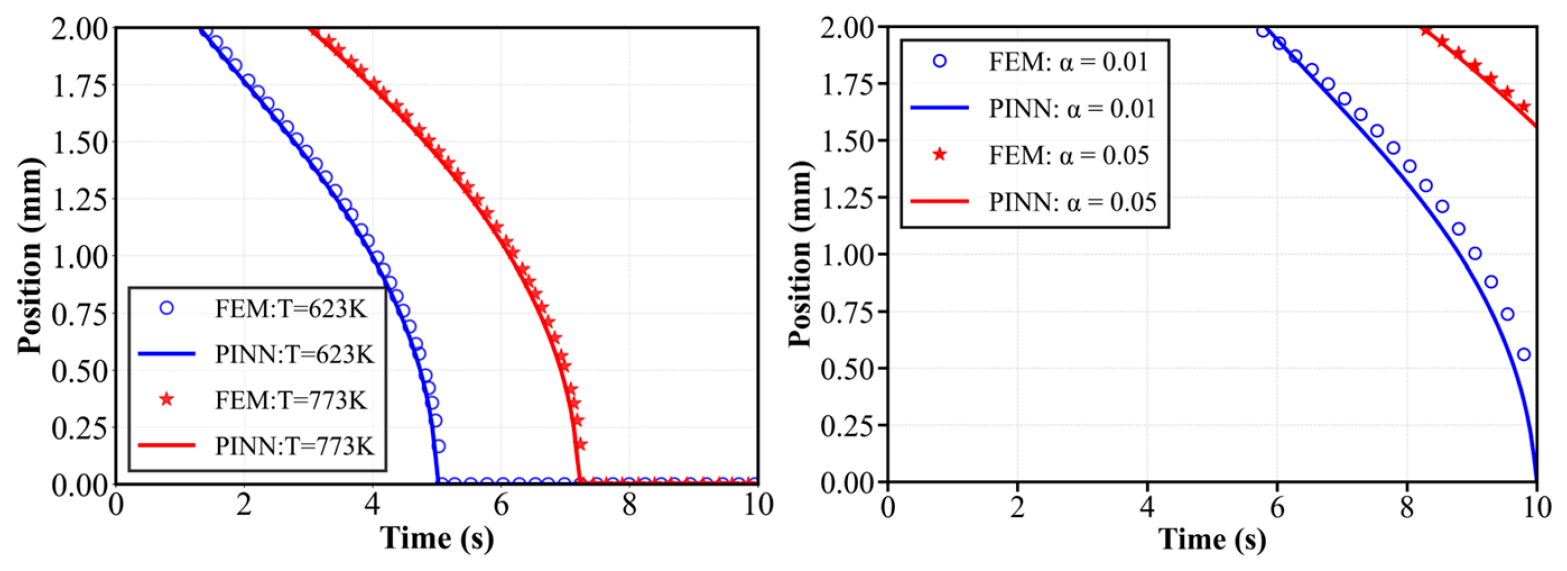
Comparison of ablation profiles (temperature and pyrolysis degree) between PINN and FEM.

As illustrated in Figure 7, the PINN solutions exhibit excellent agreement with the FEM results throughout the entire time history at characteristic locations (x = 0, 1, 2 mm). Furthermore, Figure 8 compares the spatial profiles of temperature and pyrolysis degree at the initial stage and the moment of intense reaction. The high consistency observed in both temporal and spatial domains confirms that the PINN method accurately captures the rapid dynamics of the ablation reaction.

**Case 2: Ablation Process of Type I Phenolic Material (100 W/cm^2^, N = 3)**

**Figure 9:**
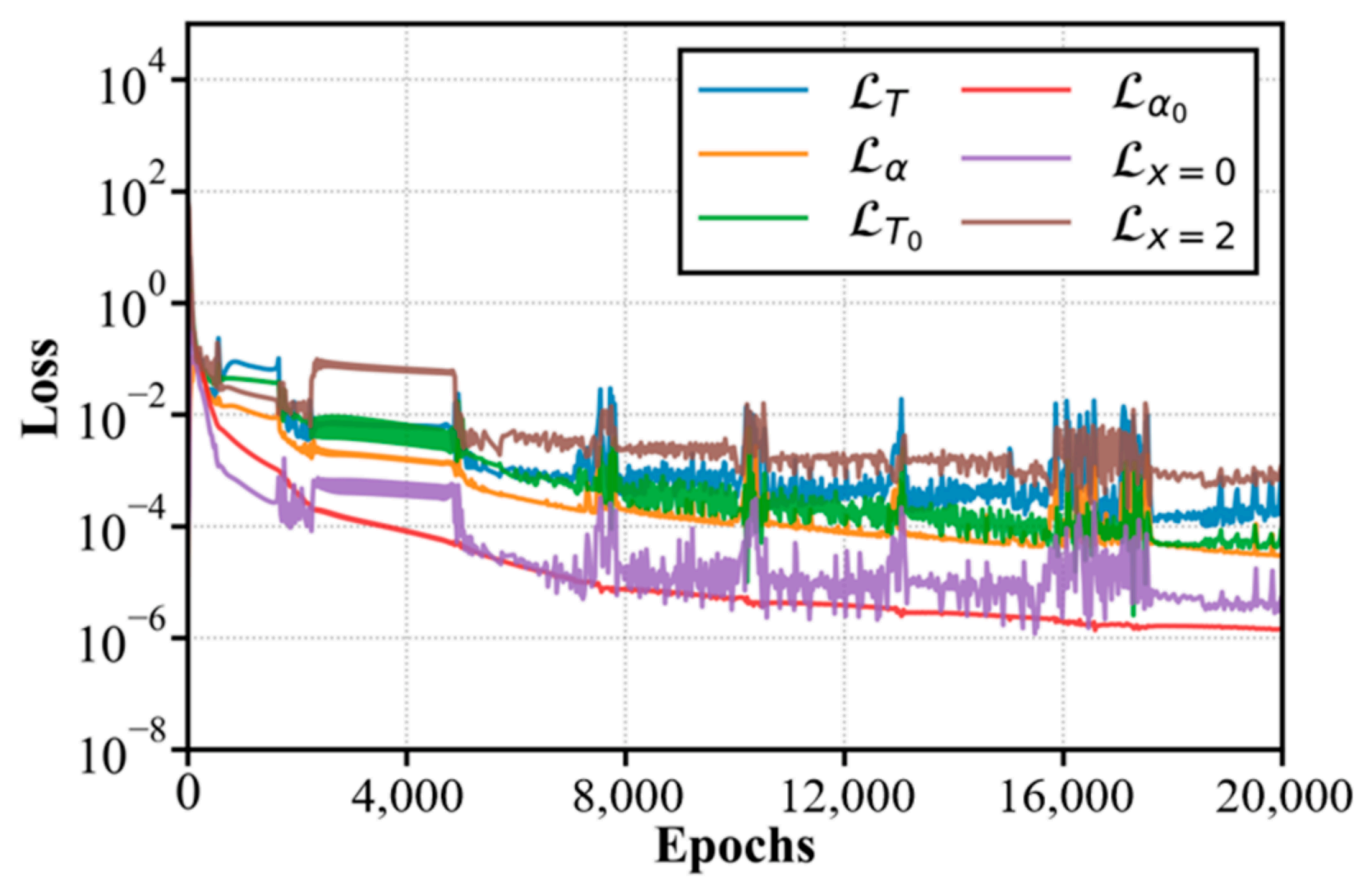
Loss function curves for Case 2.

To thoroughly demonstrate the robustness of the proposed PINN framework under different boundary constraints, an additional numerical verification Case 2 was investigated, where the top surface was exposed to a heat flux of 100 W/cm^2^. The training dynamics revealed that despite the variation in thermal loading, the overall loss function, including the coupled PDE residuals and boundary conditions, converged smoothly without severe oscillations, stabilizing at a minimum value of approximately 10^−3^~10^−6^ after 12,000 epochs, as shown in Figure 9.

**Figure 10:**
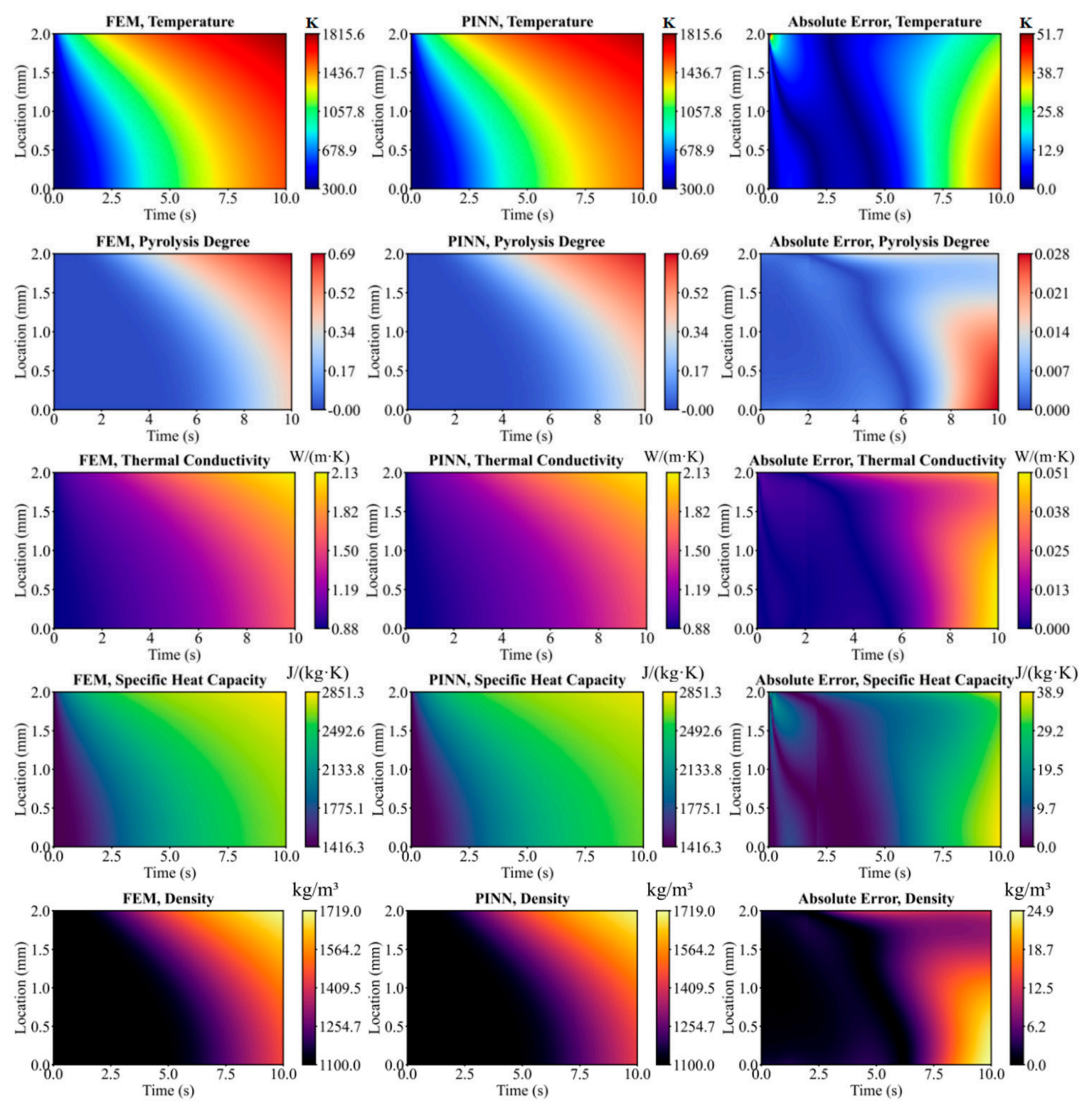
Comparison between PINN and FEM solutions (q = 100 W/cm^2^).

In Case 2, under an elevated heat flux condition of 100 W/cm^2^, the PINN solutions exhibit excellent agreement with the FEM. The relative errors for key variables, including temperature, degree of pyrolysis, and thermophysical properties, remain consistently below 4%, even during phases of intense chemical reaction, as shown in Figure 10. These results demonstrate that the proposed PINN can accurately capture the volumetric ablation behavior of phenolic resin across diverse operating conditions.

**Figure 11:**
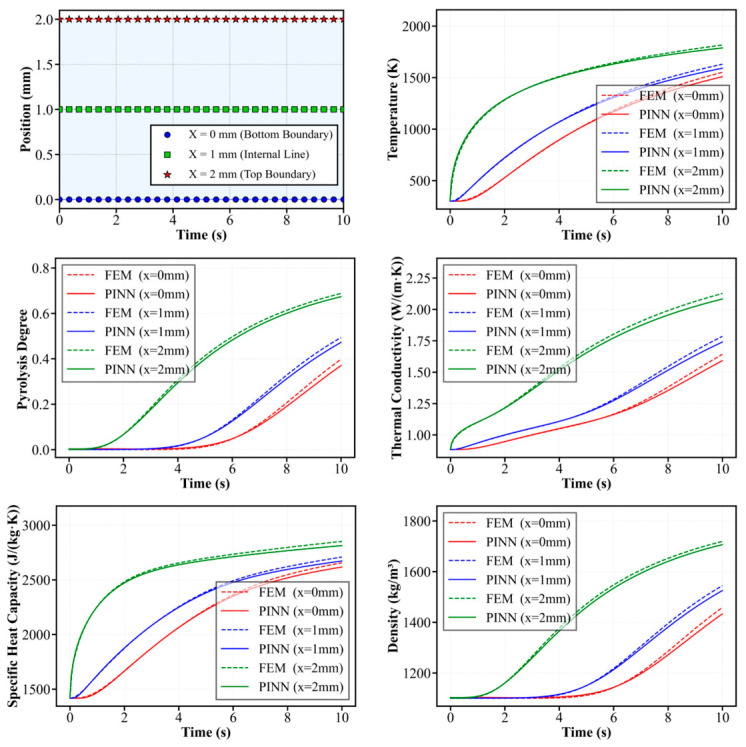
Comparison of ablation profiles (temperature and pyrolysis degree) between PINN and FEM.

By comparing the evolution profiles of temperature, degree of pyrolysis, and thermophysical properties at the boundaries (x = 0 mm and x = 2 mm) as well as the mid-plane (x = 1 mm), it is observed that the PINN and FEM solutions maintain excellent agreement across the entire spatio-temporal domain. This effectively demonstrates the numerical stability of the proposed PINN framework in both high and low temperature regions. The results are shown in Figure 11.

**Figure 12:**
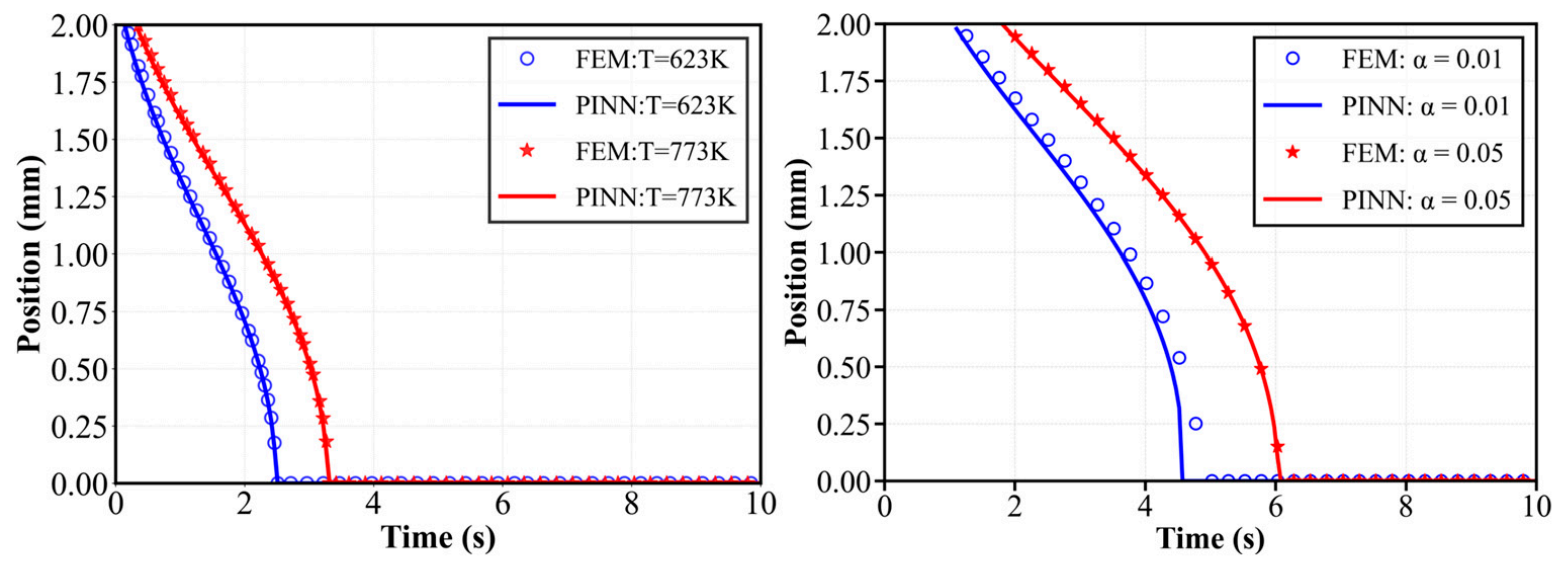
Comparison of ablation profiles (temperature and pyrolysis degree) between PINN and FEM.

Furthermore, as shown in Figure 12, a comparison is made for the temperature and degree of pyrolysis of the phenolic resin under the 100 W/cm^2^ heat flux, specifically focusing on the initial decomposition temperature (350 °C) and the intense reaction temperature (500 °C). It is evident that the PINN predictions are in near perfect agreement with the FEM solutions at both the onset location of pyrolysis and the advancing reaction front.

**Case 3: Ablation Process of Type II Phenolic Material (10 W/cm^2^, N = 3)**

**Figure 13:**
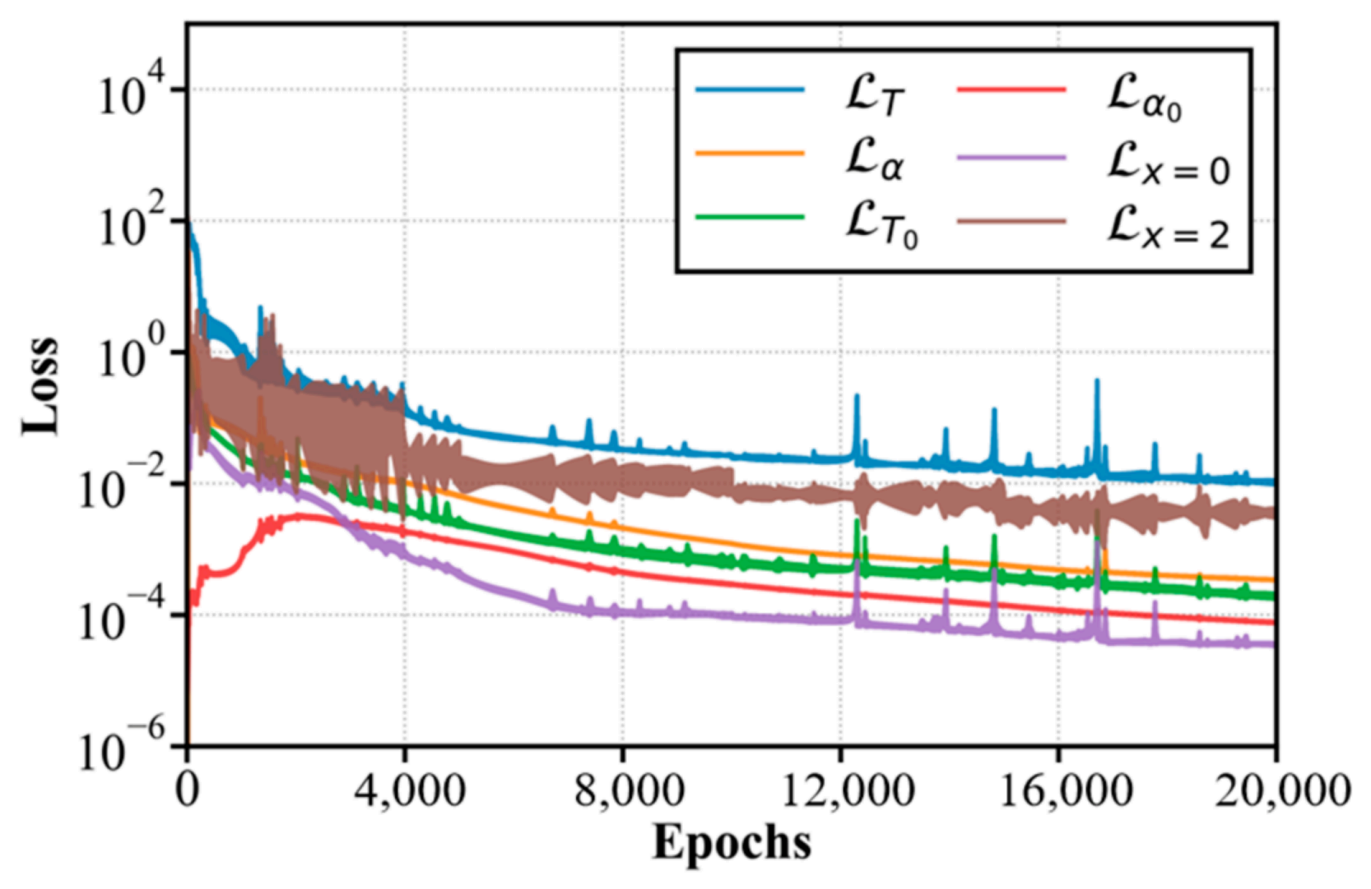
Loss function for Case 3.

The training results for the second type of phenolic resin under heat flux of 10 W/cm^2^ is shown in Figure 13. The training trajectory reveals a robust convergence of the total loss function, which effectively balances the constraints from initial conditions, boundary values, and the underlying PDEs. Ultimately, the PINN successfully completed the training process and accurately solved for the temperature field, degree of pyrolysis, and thermophysical properties.

**Figure 14:**
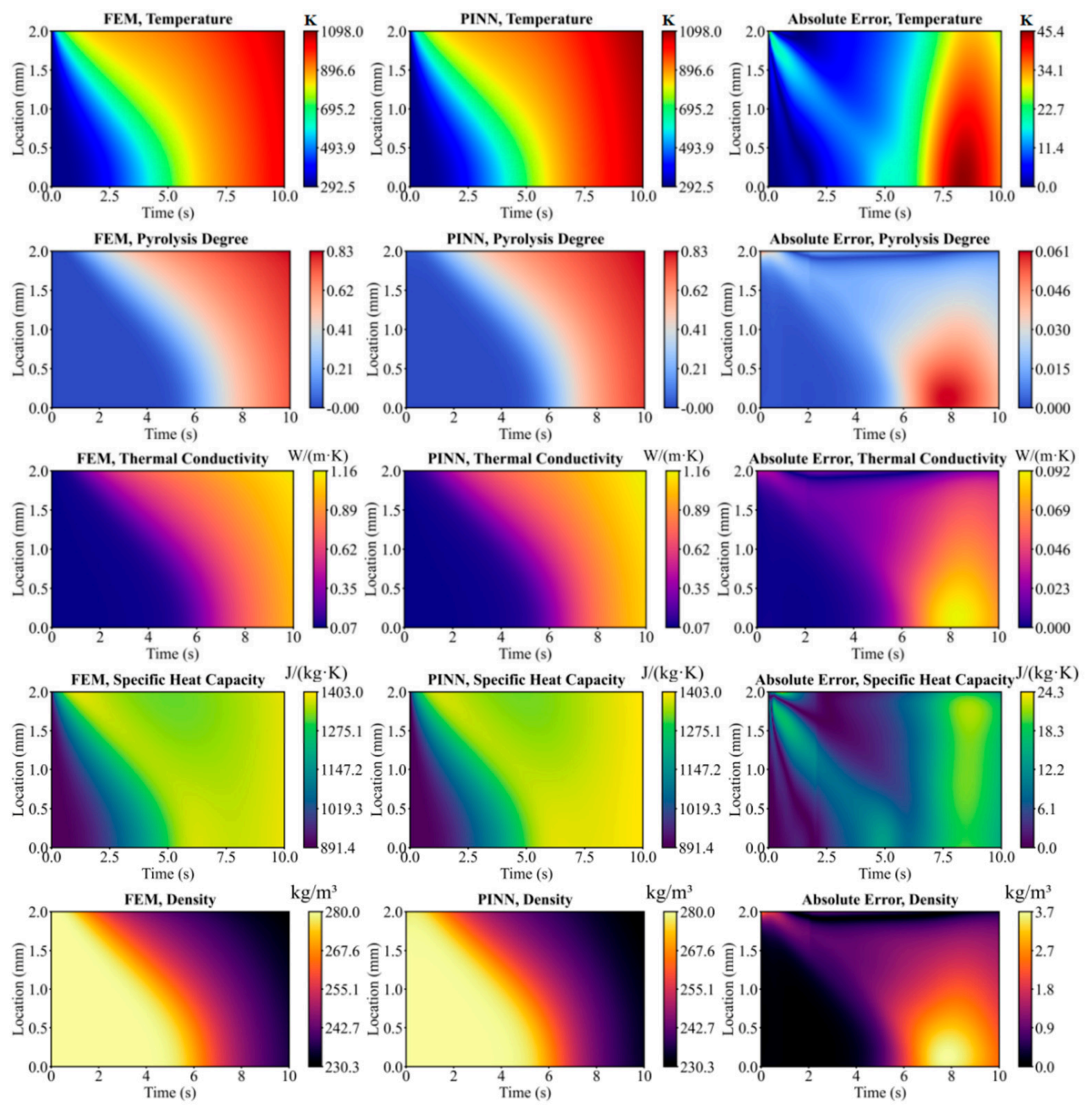
Comparison between PINN and FEM solutions (q = 10 W/cm^2^).

The PINN method demonstrated robust performance on the Phenolic II, consistently yielding stable solutions across different material properties and heat flux levels. Analysis of the results from Case 3 indicates excellent agreement between the PINN predictions and the FEM solutions, as shown in Figure 14. Throughout the entire ablation process, the relative errors for temperature, degree of pyrolysis, and thermophysical properties remain below 8%. This outcome validates the reliability of the PINN method in simulating the volumetric ablation of phenolic resin, demonstrating its superior stability and computational accuracy in handling complex coupled mechanisms such as constant heat flux, boundary radiation, and exothermic ablation reactions.

**Figure 15:**
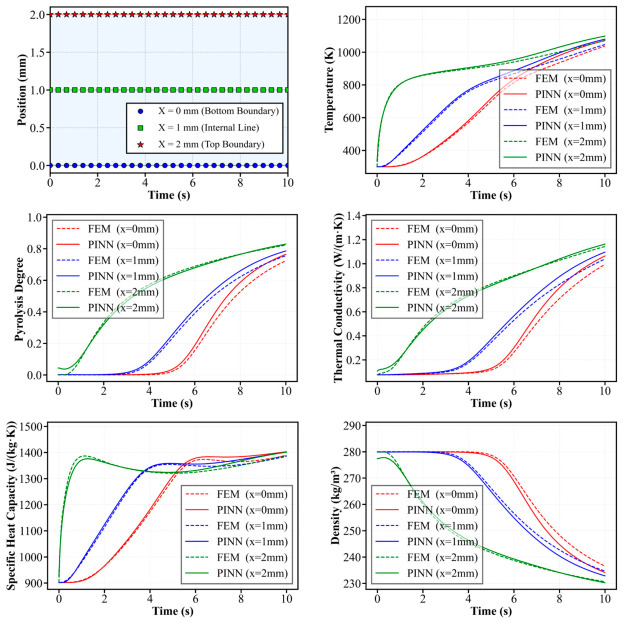
Comparison of PINN and FEM results at different locations (x = 0, 1, 2 mm).

**Figure 16:**
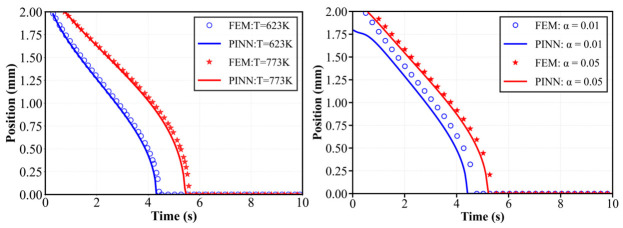
Comparison of ablation profiles (temperature and pyrolysis degree) between PINN and FEM.

As illustrated in Figure 15, the PINN solutions exhibit excellent agreement with the FEM results throughout the entire process at different locations (x = 0, 1, and 2 mm). Furthermore, Figure 16 compares the spatial profiles of temperature and degree of pyrolysis at the initial stage and the moment of intense reaction. This high consistency in both temporal and spatial domains confirms that the PINN method accurately captures the rapid dynamics of the ablation reaction and achieves precise solutions.

To quantitatively evaluate the predictive accuracy of the proposed variable-property PINN framework, Table 3 consolidates the comprehensive statistical error metrics, specifically the Mean Error, Max Error, and Root Mean Square Error (RMSE), calculated across the entire spatio-temporal domain relative to the high-fidelity FEM benchmarks. The results demonstrate exceptional precision for both the primary field variables and the reconstructed state-dependent thermal properties. Specifically, for the temperature field, the global RMSE remains strictly below 22.7750 K across all cases, with a minimum Mean Error of only 4.2009 K (Case 1). Similarly, the prediction of the pyrolysis degree (α), which spans from 0 to 1, exhibits superb accuracy, with the maximum localized discrepancy capped at 0.0610 (Case 3) and an RMSE of less than 0.0254.

Furthermore, the highly non-linear, state-dependent material properties are reconstructed with remarkable fidelity. The thermal conductivity shows negligible deviations, yielding a maximum RMSE of only 0.0397 W/(m·K) and a Mean Error under 0.0294 W/(m·K). For the specific heat capacity and density, the peak RMSE values among all test scenarios are tightly bounded at 17.8470 J/(kg·K) and 8.4635 kg/m^3^, respectively. This rigorous quantitative verification demonstrates that the developed physics-informed deep learning model is highly robust, capable of simultaneously and accurately capturing strongly coupled thermodynamic fields and severe non-linear material variations under extreme heating environments.

To further validate the physical consistency of the developed model, it is crucial to analyze how the material’s intrinsic thermodynamic properties govern the ablation intensity and directly affect the neural network’s learning performance. As tabulated in Table 2, the Type II phenolic material possesses a significantly lower thermal conductivity k compared to Type I. Mathematically and physically, this lower conductivity acts as a strong thermal barrier that severely restricts heat conduction from the exposed boundary into the deeper matrix. Consequently, even under a lower incident heat flux, severe thermal energy accumulates rapidly within a narrow sub-surface region, inducing an extremely high local temperature rise. This localized heat accumulation intensifies the chemical decomposition, resulting in a highly active and compressed pyrolysis front characterized by extremely steep spatial–temporal gradients of both the temperature T and the degree of pyrolysis alpha. From the perspective of deep learning mathematics, approximating such sharp, almost discontinuous moving reaction fronts (high spatial–temporal stiffness) poses a notorious challenge for the continuous activation functions of standard neural networks. This physical–numerical coupling perfectly explains the error distribution summarized in Table 3: For Type I material scenarios (e.g., Case 1), the higher thermal conductivity facilitates a more diffusive and spatially smoother thermal profile, allowing the PINN to easily approximate the fields with minimum errors (e.g., a temperature RMSE of only 4.5372 K). Conversely, for Type II material scenarios (e.g., Case 2 and Case 3), the intensive surface accumulation and sharp gradients increase the mathematical stiffness of the physics-informed loss landscape. This leads to slightly higher localized prediction discrepancies, with the maximum temperature error rising to 51.6650 K. This critical correlation successfully bridges the gap between material-specific properties, thermodynamic ablation intensity, and the mathematical boundaries of physics-informed deep learning solvers.

To provide an objective assessment of the computational efficiency, a direct comparison between the proposed PINN framework and the traditional FEM was conducted. Currently, the offline training of the PINN requires 19 min, which is indeed longer than the 9 min required for a single forward FEM simulation. However, the unique merit of the Physics-Informed Neural Network lies in its deployment phase. Once the network has been successfully trained, the computational cost for subsequent evaluations (online inference) or parameter inverse estimations is dramatically reduced. Unlike FEM, which requires resolving the full discretized system of equations for every new boundary condition or material parameter adjustment, the trained PINN can query any spatial–temporal coordinate almost instantaneously. This high efficiency in post-training scenarios makes the proposed framework highly competitive as a real-time surrogate modeling tool.

## 5. Conclusions

This study proposes an innovative computational framework based on Physics-Informed Neural Networks (PINNs) to address the multi-physics coupling problems of bulk ablation in phenolic resin composites under extreme high-temperature environments.

(i) Embedded solution for strong thermo-chemical coupling mechanisms: This framework breaks the reliance of traditional numerical methods on the linearization or decoupling of physical parameters. By directly encoding the governing equations into feedforward neural networks, it achieves a fully coupled solution for the heat conduction equation with internal heat sources and the pyrolysis kinetics equation. Notably, the strong non-linearity arising from the significant changes in thermophysical parameters with temperature and degree of pyrolysis is addressed by the PINNs. By enabling the real-time dynamic evolution of material properties at all collocation points, the complex physicochemical responses inside the material are accurately captured.

(ii) Mesh-free Architecture and Robustness Optimization: To overcome the issue of mesh distortion resulting from phase changes and severe gradients in ablation problems, the proposed PINN utilizes a mesh-free architecture. This approach successfully liberates the solution process from the constraints of grid discretization. Additionally, nondimensionalization is employed to address the ill-conditioning of the loss function, thereby significantly improving convergence stability for stiff problems.

(iii) High-Fidelity Prediction and Surrogate Model Potential: Numerical verification confirms the model’s accuracy, with the maximum relative error for temperature, degree of pyrolysis, and evolving material properties remaining within 8.0% of the FEM reference solutions. This confirms the high fidelity of the PINN framework in handling non-linear ablation problems, laying a solid foundation for building deep learning surrogate models to facilitate the rapid design and evaluation of thermal protection systems for fiber-reinforced phenolic resin matrix composites.

Despite its high accuracy, several limitations of the current PINN model must be addressed to define its engineering boundaries. First, the framework relies on numerical cross-verification against FEM benchmarks and currently lacks direct calibration using in situ experimental diagnostics. Second, the solver is restricted to a 1D spatial domain, neglecting multi-dimensional (2D/3D) thermal gradients. Lastly, extreme-regime physics, specifically chemical–mechanical surface recession (moving boundaries) and porous pyrolysis gas transport (internal convective cooling), are simplified or omitted. Future research will focus on transitioning to multi-dimensional, moving-boundary formulations coupled with porous media flow to enhance the fidelity.

## Figures and Tables

**Table 1:** Boundary conditions and reaction kinetics.

Material	Case	Boundary Condition (W/cm^2^)	N	Time (s)
phenolic resin I	1	31	1	10
	2	100	3	10
phenolic resin II	3	10	3	10
	1-A	20	4	10

**Table 2:** Parameters of the phenolic ablation model in Equation (17) [29].

Parameter	Phenolic Resin I	Phenolic Resin II
Virgin material density $\rho_{b}\left({\mathrm{kg}/m}^{3}\right)$	1100	280
Char density $\rho_{p}\left({\mathrm{kg}/m}^{3}\right)$	2000	220
$\mathrm{Specific}\;\mathrm{heat}\;\mathrm{capacity}\;\mathrm{of}\;\mathrm{the}\;\mathrm{virgin}\;\mathrm{material}\;C_{b}\left(J/\mathrm{kg}\cdot K\right)$	1090 + 1.09 T	900 + 1.0 (T−298)
$\mathrm{Specific}\;\mathrm{heat}\;\mathrm{capacity}\;\mathrm{of}\;\mathrm{the}\;\mathrm{charred}\;\mathrm{material}\;C_{p}\left(J/\mathrm{kg}\cdot K\right)$	880 + 1.02 T	700 + 0.8 (T−298)
$\mathrm{Thermal}\;\mathrm{conductivity}\;\mathrm{of}\;\mathrm{the}\;\mathrm{virgin}\;\mathrm{material}\;k_{b}\left(J/m\cdot s\cdot K\right)$	8.0 + 2.76 × 10^−3^ T	0.06 + 5 × 10^−5^ T
$\mathrm{Thermal}\;\mathrm{conductivity}\;\mathrm{of}\;\mathrm{the}\;\mathrm{charred}\;\mathrm{material}\;k_{p}\left(J/m\cdot s\cdot K\right)$	9.6 + 8.42 × 10^−3^ T	0.5 + 8.0 × 10^−4^ T
$\mathrm{Activation}\;\mathrm{energy}\;\mathrm{of}\;\mathrm{phenolic}\;\mathrm{resin}\;\mathrm{pyrolysis}\;E_{a}\left(J/\mathrm{mol}\right)$	7.88 × 10^4^	7.11 × 10^4^
Pre-exponential factor $J_{0}\left({\mathrm{kg}/m}^{3}\cdot s\right)$	1.94 × 10^5^	3.36 × 10^6^
Heat of pyrolysis $H_{v}\left(J/\mathrm{kg}\right)$	4.187 × 10^5^	2 × 10^5^

**Table 3:** Summary of error analysis.

Variable	Error	Case1	Case2	Case3
**Temperature** **(K)**	Mean Error	4.2009	13.4590	18.4090
	Max Error	29.8780	51.6650	45.4280
	RMSE	4.5372	17.9780	22.7750
**A**	Mean Error	0.0009	0.0064	0.0195
	Max Error	0.0043	0.0277	0.0610
	RMSE	0.0011	0.0094	0.0254
**Thermal Conductivity** **W/(m·K)**	Mean Error	0.0015	0.0125	0.0294
	Max Error	0.0085	0.0505	0.0919
	RMSE	0.0016	0.0187	0.0397
**Specific Heat Capacity** **J/(kg·K)**	Mean Error	3.6219	13.9240	9.8168
	Max Error	32.4040	38.9070	24.3430
	RMSE	4.0378	17.8470	11.8380
**Density** **(kg/m^3^)**	Mean Error	0.7665	5.7994	1.1703
	Max Error	3.9131	24.9250	3.6589
	RMSE	0.9945	8.4635	1.5261

**Figure A1:**
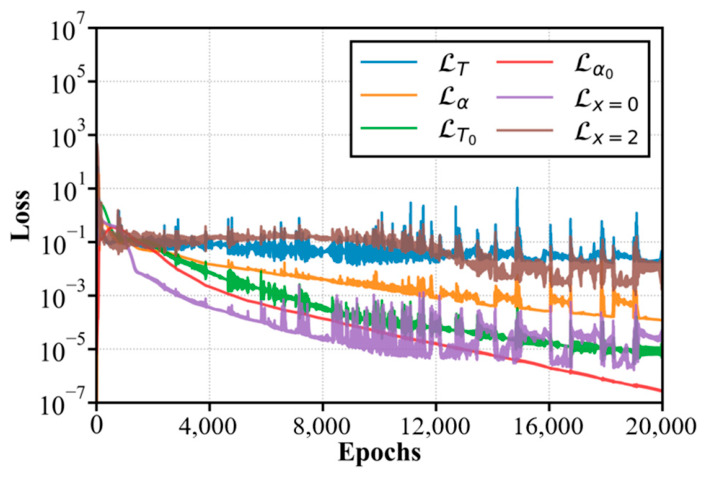
Loss function curves for Case 1–4.

**Figure A2:**
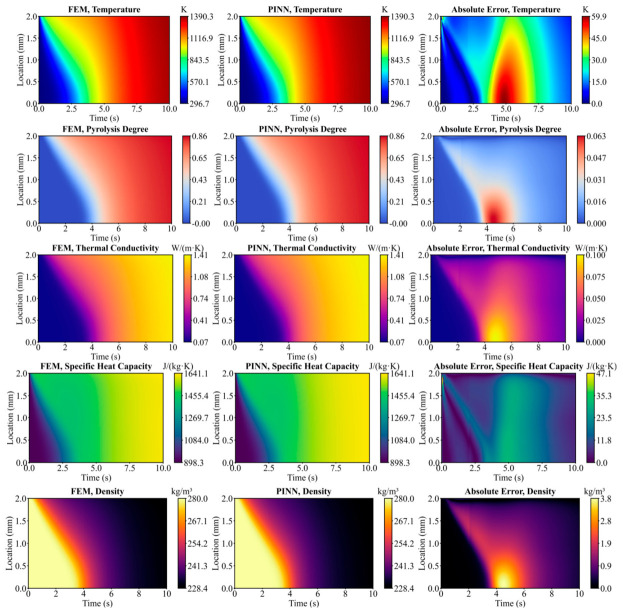
Comparison between PINN and FEM solutions (q = 20 W/cm^2^).

**Figure A3:**
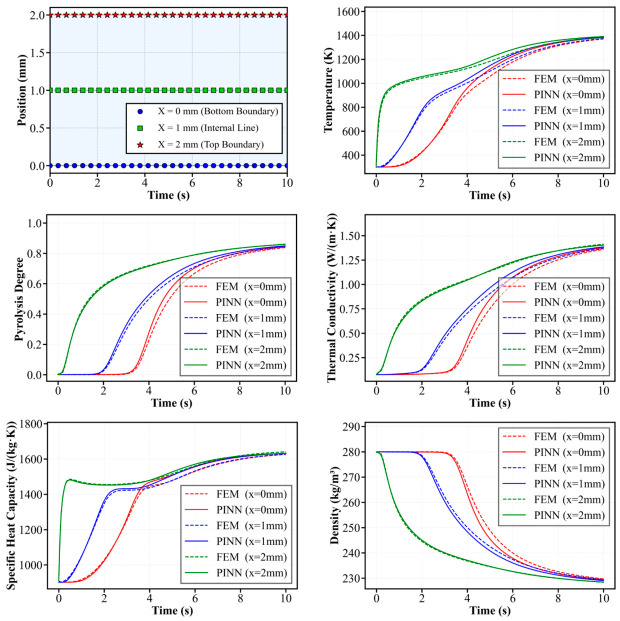
Comparison of ablation profiles (temperature and pyrolysis degree) between PINN and FEM.

**Figure A4:**
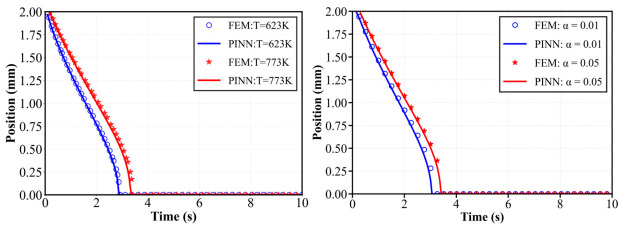
Comparison of ablation profiles (temperature and pyrolysis degree) between PINN and FEM.

## Data Availability

The data presented in this study are available on request from the corresponding author.

## Appendix A

### Appendix A.1. Grid-Free Collocation Point and Network Architecture Independence Study

In grid-free Physics-Informed Neural Network computing, proving that the converged prediction is independent of the spatial–temporal collocation point density and neural layer hyperparameters is critical for establishing algorithmic robustness. To this end, a systematic sensitivity study was executed. We tested various combinations of domain collocation points and network sizes. Table A1 presents the converged error profiles and corresponding computational costs.

**Table A1:** Summary of error analysis.

Config.	Collocation Points	Network Size	Relative Temp Error	Average Training Time
1.	5000.	5 × 128	1.42%	~8 min
2.	10,000.	5 × 128	0.84%	~13 min
3.	20,000.	5 × 128	0.54%	~19 min
4.	40,000.	5 × 128	0.52%	~31 min
5.	20,000.	4 × 64	2.15%	~15 min
6.	20,000.	6 × 256	0.51%	~29 min

The quantitative trade-offs reported in Table A1 justify the physical baseline configurations employed throughout this study. When the collocation point is restricted below 10,000 (Config.1 and Config.2), the localized Arrhenius-driven pyrolysis front is inadequately resolved, raising the maximum relative error above 1.0%. However, as scales to 20,000 (Config.3), the error declines to a stabilized plateau within the highly acceptable 0.54% margin. Further doubling the spatial sampling points to 40,000 (Config.4) merely increases training latency with no meaningful gains in fidelity. Similarly, comparing Config.3, Config.5, and Config.6 confirms that a five-layer network with 128 hidden neurons per layer provides the optimal balance; deeper and wider architectures suffer from severe diminishing returns, causing unnecessary computational overhead. These outcomes confirm that our numerical predictions are highly robust and fully independent of hyperparameter variations beyond the selected baseline.

### Appendix A.2. Finite Element Verification and UMATHT Subroutine Architecture

To construct a rigid numerical benchmark for the validation of the Physics-Informed Neural Network (PINN), a high-fidelity continuum finite element model was set up in Abaqus/Standard using DC3D8 (8-node linear thermal brick) elements. Due to the severe thermal gradients induced by localized high-power energy deposition and rapid mass loss at the ablation front, the solution accuracy depends heavily on localized grid density. A grid convergence study was executed. The numerical results are outlined in Table A2.

**Table A2:** FEM grid sensitivity evaluation.

FEM.	Minimum Element Size	Peak Temperature	Calculation Time
1.	0.5 mm	1723.4 K	~2 min
2.	0.2 mm	1786.7 K	~6 min
3.	0.1 mm	1815.6 K	~9 min
4.	0.05 mm	1817.3 K	~12 min

As shown in Table A2, FEM.3 was chosen as our baseline reference because further spatial or temporal refinement (FEM.4) alters the peak ablation parameters by less than 0.1%, while exponentially increasing the computational cost.

The volumetric ablation behavior of phenolic resin is computed via a customized UMATHT user subroutine. For each integration point at each kinematic increment, Abaqus passes in the current temperature T, temperature gradient ΔT, and the internal state array STATEV.

The mathematical formulation evaluated within the routine loop follows the flow layout below:

The degree of material pyrolysis α (stored as STATEV(1)) is mapped using the instantaneous temperature T.

(A1) $$\Delta \alpha =Ae^{-E_{a}/RT}{\left(1-\alpha \right)}^{N}\cdot \Delta t$$

(A2) $$\alpha_{\mathrm{new}}=\alpha_{\mathrm{old}}+\Delta \alpha$$

The internal volumetric energy density U and its temporal Jacobian tangent derivative DUDT (∂U/∂T) are evaluated by accounting for both sensible heat and chemical reaction enthalpies $\Delta H_{pyro}$:

(A3) $$U=\rho \left(\alpha \right)\int_{T_{0}}^{T}C_{p}\left(\theta ,\alpha \right)d\theta +\left(1-\alpha \right)\rho_{0}\Delta H_{pyro}$$

(A4) $$DUDT\left(i,j\right)=-k_{ij}\left(T,\alpha \right)$$

The spatial heat flux vector fi (FLUX(i)) and its spatial derivative matrix DFLUX(i,j) are updated to define the instantaneous anisotropic thermal conductivities:

(A5) $$FLUX\left(i\right)=-k_{ij}\left(T,\alpha \right)\cdot \frac{\partial T}{\partial x_{j}}$$

(A6) $$DFLUX\left(i,j\right)=-k_{ij}\left(T,\alpha \right)$$

This comprehensive formulation guarantees that all highly non-linear state-dependent variations are properly evaluated inside the residual force vector of the FEM global solver, providing an absolute validation baseline for the PINN methodology.

### Appendix A.3. Case 1-A: Ablation Process of Type Phenolic II Material (20 W/cm^2^, N = 4)

To further assess the generalizability of the proposed PINN framework, an additional numerical verification case 1-A was conducted using Phenolic II subjected to a heat flux of 20 W/cm^2^. While the distinct thermophysical properties and modified pyrolysis reaction order result in a significantly more intensive ablation process, the loss function maintains an orderly descent overall. To evaluate the optimization efficiency and training stability of the developed PINN framework, the detailed history of the loss minimization is illustrated in Figure A1. As the training epochs progress, all six loss components exhibit a smooth, non-oscillatory decay, confirming the effectiveness of the chosen optimization strategy and dynamic weight balances. Ultimately, the loss curves demonstrate consistent convergence behaviors, stably leveling off at distinct steady-state magnitudes. Specifically, the boundary and initial constraint losses achieve a high-precision minimum around a magnitude of 10^−6^ due to their explicit supervisory signals, while the heavily coupled, non-linear PDE residual terms stably converge within a bounded range of 10^−3^ to 10^−5^. This convergence profile validates that the network parameters are successfully optimized without falling into divergent numerical oscillations.

Regarding the physical predictions, the model was tasked with solving five distinct types of variables associated with the pyrolysis reaction. The intensified reaction dynamics introduce local fluctuations during the solving process, leading to a marginal increase in point-wise discrepancies compared to previous cases. Despite these fluctuations, the predicted spatio-temporal contours of temperature, degree of pyrolysis, and other key thermophysical properties maintain a high degree of fidelity to the FEM solutions. The maximum relative error across all five variables remains strictly within 8%, confirming the model’s robustness even under extreme ablative conditions.

As shown in Figure A3 and Figure A4, the PINN predictions exhibit exceptional fidelity to the FEM solutions. The model accurately solves for the temporal evolution of each physical quantity at various locations throughout the entire process. Furthermore, the framework successfully resolves the complex spatial distributions of temperature and the degree of pyrolysis, particularly during the critical transition from the initial heating phase to the intense reaction stage. This robust spatio-temporal consistency across multiple cases established that the proposed PINN can reliably capture the highly non-linear and rapid dynamics of the ablation process.
